# Event-Based 3D Motion Flow Estimation Using 4D Spatio Temporal Subspaces Properties

**DOI:** 10.3389/fnins.2016.00596

**Published:** 2017-02-06

**Authors:** Sio-Hoi Ieng, João Carneiro, Ryad B. Benosman

**Affiliations:** Institut National de la Santé et de la Recherche Médicale, UMRI S 968; Sorbonne Université, University of Pierre and Marie Curie, Univ Paris 06, UMR S 968; Centre National de la Recherche Scientifique, UMR 7210, Institut de la VisionParis, France

**Keywords:** neuromorphic vision, event-based sensing, scene flow, 3D point clouds, motion estimation, motion from structure

## Abstract

State of the art scene flow estimation techniques are based on projections of the 3D motion on image using luminance—sampled at the frame rate of the cameras—as the principal source of information. We introduce in this paper a pure time based approach to estimate the flow from 3D point clouds primarily output by neuromorphic event-based stereo camera rigs, or by any existing 3D depth sensor even if it does not provide nor use luminance. This method formulates the scene flow problem by applying a local piecewise regularization of the scene flow. The formulation provides a unifying framework to estimate scene flow from synchronous and asynchronous 3D point clouds. It relies on the properties of 4D space time using a decomposition into its subspaces. This method naturally exploits the properties of the neuromorphic asynchronous event based vision sensors that allows continuous time 3D point clouds reconstruction. The approach can also handle the motion of deformable object. Experiments using different 3D sensors are presented.

## 1. Introduction

### 1.1. Scene flow

The motion of 3D structures is an important information to extract from a scene to build geometric and dynamic descriptions of its content. Such information is also essential to a large set of vision applications such as: virtual reality synthesis, scene segmentation and autonomous navigation. Scene flows are vector fields that map points of a 3D structure to their instantaneous velocity vectors. Because of this close relationship, estimating the scene flow usually implies to estimating the structure and vice-versa.

The Structure From Motion (SFM) is one of the classical computer vision problems that have been largely studied during the past few decades by the machine vision community (Maybank, 1993). However, SFM's high vulnerability to images' noise and to camera calibration errors raised questions regarding its applicability in real-world scenarios (Tomasi and Zhang, 1995). Currently, with the increasing demand for realistic and high definition 3D content, many ready-to-use sensors are now able to provide dense 3D points clouds in real-time (such as: laser range-finders, structured light vision sensors,…). These devices allow to decouple the structure reconstruction from the motion estimation and to focus the effort on motion extraction and its characterization.

To achieve dense scene flow estimation, state-of-the-art techniques estimate depth maps and compute optical flows for each camera separately. In a second stage they combine both to estimate the 3D flow. This approach parametrizes the motion problem on the image plane, i.e., in 2D and is the most commonly found in the existing literature (Vedula et al., 1999; Zhang et al., 2001; Isard and MacCormick, 2006; Wedel et al., 2011). A 2D parametrization is however more prone to discontinuities since a smooth 3D signal may be projected into a discontinuous 2D signal due to occlusions.

In Basha et al. (2013), the depth map and the optical flow are solved simultaneously rather than in a sequential manner, as authors argue, for a better coupling between spatial and temporal information. In Hadfield and Bowden (2014) and Park et al. (2012), the motion flow is extracted and refined directly from the 3D points clouds by using particle filtering or tensor voting techniques. Optical flows are only estimated for comparison purposes or for initial scene flow estimation.

A second requirement for obtaining dense flow estimation is to introduce some form of regularization. For that purpose, one recurrent hypothesis is to assume local rigid body motion and therefore induce local constant velocity, i.e., points on a non-deformable surface will have the same velocity. Regularization is often performed by minimizing an energy function with variational formulations (Zhang et al., 2001; Min and Sohn, 2006; Huguet and Devernay, 2007). Energy minimization has proven to be a successful technique for both 2D and 3D flow parametrization. It is however computationally greedy and it makes it difficult to achieve real-time estimation without embedding a dedicated powerful computational unit (e.g., GPU). Scene flow can also be computed from local descriptors of reconstructed surfaces such as surfel that encodes the local geometry and the reflectance information of the shapes (Carceroni and Kutulakos, 2002). Motion is then estimated in an integrative manner by matching descriptors over time. Several authors adopted the same idea of addressing the scene flow as a problem of characterization and tracking 3D surfaces over time. Varanasi et al. (2008) proposes to describe and track the surfaces by sparse features matching and extend this to a dense estimation using smoothing operations based on the Laplacian diffusion. Patch based techniques have also been used in Popham et al. (2010) and Cagniart et al. (2010) to split complex surfaces into simpler ones. Their matching and relative pose estimation for each patch allows to estimate the scene flow densely.

This paper introduces a new solution to estimate scene flow using properties of 4D (3D space+time) spaces without the need to use luminance. We will show that the use of the time allows to go beyond the conventional framework that relies on the combined use of luminance and depth information (Herbst et al., 2013). The paper is initially intended to operate on high temporal resolution 3D depth information output from a binocular neuromorphic event-based camera stereo rig. As introduced in Rogister et al. (2012) and Carneiro et al. (2013), event-based cameras allow to estimate depth and produce 3D point clouds at unprecedented accuracy (>1 kHz in real-time) at very low computational and energy cost using conventional processing hardware. We will show that the method can be used even in the case of lower temporal resolution and it can be applied to any 3D data such as the ones output from: RGB-D cameras (Khoshelham and Elberink, 2012), time-of-flight range-imaging sensors (Hansard et al., 2012), laser range finder and even conventional camera based systems that are also able to provide robust 3D reconstructions with a reasonable accuracy using optimized implementations. It is however important to notice that beyond the heavy computational and energy requirement, all these techniques rarely exceed frame rates beyond 90 Hz.

We will then show that the use of timed 4D spaces (3D space + time) allow to derive more efficient techniques than state of art techniques. The method assumes locally non-deformable spatiotemporal surfaces swept by 3D moving structures. We show that under such hypothesis, the velocity estimation is reduced to a one dimensional search over ℝ, the set of real numbers, and the dense estimation is directly achieved using local spatiotemporal planes. An additional advantage is its ability to determine velocities collinear to moving edges assuming it is possible to identify local 3D structures across the trajectory. This work can be seen as a generalization of the previous work on the event-based estimation of 2D visual motion flow (Benosman et al., 2014) to higher dimensional spaces.

### 1.2. Asynchronous event-based vision

Biological retinas do not encode visual scenes as collection of static frames, but rather as a continuous stream of asynchronous spikes. Neuromorphic vision sensors replicate partially this mechanism by encoding visual information with high temporal resolution asynchronous streams of events. Since the pioneering work of Mahowald (1992) that built the first retina on silicon, several major improvements have been made for what is now refered to as the “neuromorphic silicon retinas.” One of the most important achievements is the Dynamic Vision Sensor (DVS) (Lichtsteiner et al., 2008), a 128 × 128 pixel resolution sensor which encodes light intensity changes into a stream of asynchronous events. Each pixel responds independently to contrast changes producing ON and OFF events (respectively to increase or decrease in light intensity) at microsecond resolution.

Posch et al. designed the Asynchronous Time-based Imaging Sensor (ATIS) (Posch et al., 2011), a 302 × 240 pixel resolution sensor which measures absolute luminance information when a contrast change event occurs. The sensor provides a 143 dB dynamic range gray-level information asynchronously encoded as the temporal difference of two exposure measurement events. Its typical temporal accuracy is around 1μs. The reader can refer to Delbrück et al. (2010) for a complete review of the existing neuromorphic visual sensors.

## 2. Materials and methods

### 2.1. Scene flow parametrization

**Figure 1:**
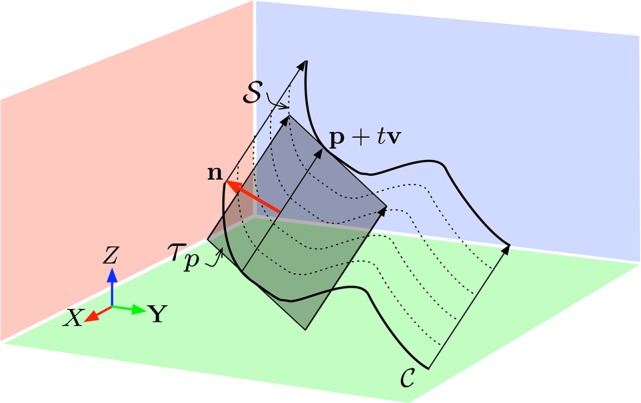
**The non-deformable surface hypothesis allows to assume the velocity v is locally constant**. The surface S swept by the edge C in the direction **v** is a ruled surface whose tangent plane τ_*p*_ at **p** allows to recovering **v** if sufficient geometric constraints can be derived. The vector **n** is the normal to τ_*p*_.

We define a 3D event as a 4-components vector (*x, y, z, t*)^*T*^. It can be increased to 5 components if the luminance information is available. Let us consider a smooth edge C which can be assumed planar within a small enough spatial neighborhood. If the velocity of C is constant, then as time increases, the edge generates a ruled surface S in the direction of the velocity **v**. The surface can be algebraically defined by the equation:

(1) $$\begin{array}{l} S:{\mathbb{R}}^{3}\times \;{\mathbb{R}}^{+}\;\to \;\mathbb{R}\\ \;(x,\;y,\;z,\;t)\mapsto S(p+tv)=0 \end{array}$$

where p∈C. Figure 1 shows an illustration of such ruled surface.

The velocity vector is according to Equation (1) the directrix of the ruled surface swept by the edge, hence the estimation of **v** is equivalent to determining the surface's directrix. In addition to Equation (1), if the surface is smooth (i.e., of class $C^{1}$ at least), we get a second equation satisfied by **v**:

(2) $${\left(\nabla S\right)}^{T}v=0,$$

because the directrix **v** is contained in the tangent plane $T_{p}$ (Sommerville, 1934). ∇S refers to the gradient of S. Only the direction of **v** can be deduced from the two scalar equations since **v** has 3 components. Its norm can be set arbitrarily to 1. To determine the exact amplitude, additional constraints are required. A possible way to estimate amplitudes is to apply a shape registration technique, since the velocity vector is the vector joining the two consecutive positions of the shape when it moves. We then propose to estimate the velocity in a two steps operation:

1. a local fitting of a smooth surface to the 3D points clouds is operated to derive as much equations similar to (1) and (2) as possible,
2. **v** is then estimated from the equations established in step 1 by adding a shape registration algorithm.

To get enough equations to estimate **v**, we propose to study three surfaces derived from S. Let $S_{1}$, $S_{2}$ and $S_{3}$ be respectively the surfaces built from Equation (1) in each coordinate frame (*X, Y, T*), (*Y, Z, T*), and (*Z, X, T*). Because of the constant velocity hypothesis, we get three surfaces with implicit equations of the form:

(3) $$S_{k}(i,\;j,\;t)=S_{k}\left(\left(\begin{array}{c} p_{i}\\ p_{j}\\ 0 \end{array}\right)+\;t\;\left(\begin{array}{c} v_{i}\\ v_{j}\\ 1 \end{array}\right)\right)=0,$$

where (*i, j*) is any pair of elements in {(*x, y*), (*y, z*), (*z, x*)} and *k* indexes the *k*^*th*^ element of this list e.g., if *k* = 1, (*i, j*) = (*x, y*). This means we are working with the *x, y* and *t* components of *S*.

These surfaces are also ruled surfaces of respective directrices (*v_x_*,*v_y_*, 1)*^T^*, (*v_y_*,*v_z_*, 1)*^T^* and (*v_z_*,*v_x_*, 1)*^T^* and their generatrices are the restrictions of C to (*X*, *Y*, *T*), (*Y*, *Z*, *T*) and (*Z*, *X*, *T*). For the same reason, given Equation (2), we can establish for each $S_{k}$ the equation:

(4) $${\left(\nabla S_{k}\right)}^{T}\left(\begin{array}{c} v_{i}\\ v_{j}\\ 1 \end{array}\right)=\frac{\partial S_{k}}{\partial i}v_{i}+\frac{\partial S_{k}}{\partial j}v_{j}+\frac{\partial S_{k}}{\partial t}=0.$$

**Figure 2:**
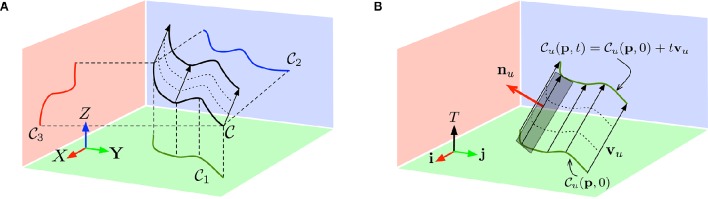
**(A)** A 3D edge C moving at constant velocity **v** is projected as 2D curves in each of the three planes (*O, X, Y*), (*O, Y, Z*) and (*O, Z, X*). **(B)** Each of the projected curve *C*_*u*_ for 1 ≤ *u* ≤ 3 is also moving at constant speed **v***_u_* = (*v_i_*, *v_j_*, 1)*^T^* in the coordinate frames (*ijT*) ((*i, j*) being any element in the set {(*x, y*), (*y, z*), (*z, x*)}) and is sweeping a ruled surface as t increases.

As illustrated by Figure 2, we now have three geometric constraints, which can be rearranged into a matrix form:

(5) $$\underset{M}{\underset{︸}{\left(\begin{array}{ccc} S_{1,x} & S_{1,y} & 0\\ 0 & S_{2,y} & S_{2,z}\\ S_{3,x} & 0 & S_{3,z} \end{array}\right)}}\;v=\;-\;\left(\begin{array}{c} \partial S_{1}/\partial t\\ \partial S_{2}/\partial t\\ \partial S_{3}/\partial t \end{array}\right),$$

with the convention that $S_{k,x}$ (respectively *y, z*) is the partial derivative with respect to *x* (respectively *y*,*z*). To determine **v**, the ideal case would be to have *M* invertible i.e., it is full ranked. There is no obvious way to tell from the general expression of *M*.

### 2.2. Plane approximation

Solving Equation (5) for **v** cannot be done without knowing the analytic equations of $S_{k}$, so we propose to apply a local plane fitting to establish the matrix *M*. The choice of a plane instead of a more complex surface is motivated by the fitting simplicity and its computational cost even though planes give rise to rank-2 matrices *M*, as it will be shown further.

Let Π_1_, Π_2_, and Π_3_ be the planes that are fitted locally to the surfaces $S_{1}$, $S_{2}$, and $S_{3}$ respectively. They then can be locally expressed using the plane's implicit equation as:

(6) $$S_{k}(i,j,t)={\Pi_{1}}^{T}\left(\begin{array}{c} i\\ j\\ t\\ 1 \end{array}\right)=0,$$

where ${\Pi_{k}}^{T}=(a_{k},\;b_{k},\;c_{k},\;d_{k})$, for 1 ≤ *k* ≤ 3.

If we derive Equation (6) with respect to each of the spatial and temporal components and for each $S_{k}$, then Equation (5) becomes

(7) $$\left(\begin{array}{ccc} a_{1} & b_{1} & 0\\ 0 & a_{2} & b_{2}\\ b_{3} & 0 & a_{3} \end{array}\right)\;v=-\left(\begin{array}{c} c_{1}\\ c_{2}\\ c_{3} \end{array}\right).$$

### 2.3. Rank of *M*

Under the local plane hypothesis we previously made, it is possible to determine the rank of *M*. For that purpose, we assume the hypothesis that the edge C is a straight line segment defined by a point **p**_0_, a direction vector **u**, and parametrized by a real α:

(8) $$p\in C\Rightarrow p=p_{0}+\alpha u,$$

and the equation of S is changed into:

(9) $$S(p,v,t)=S(p_{0}+\alpha u+tv)=0.$$

**Figure 3:**
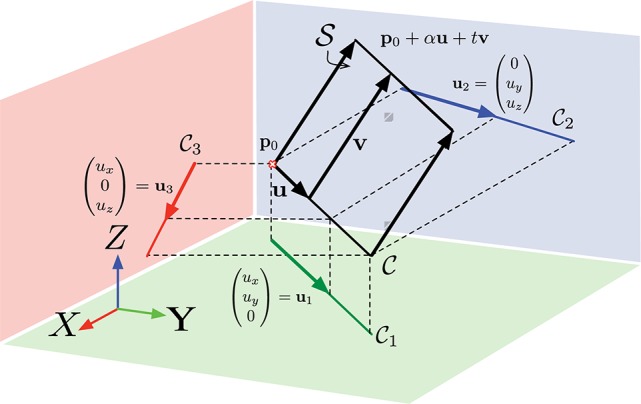
**The local fitting of a plane to the point cloud allows approximating the plane tangent to the surface swept by an edge as it moves**. If the velocity is constant, the so built surface is called ruled surface and the velocity vector **v** is its directrix. To estimate **v**, it is, up to approximation errors, equivalent to working on the tangent plane.

Figure 3 depicts the case where C is a line and the resulting ruled surface S, obtained by sweeping lines in the direction of **v** is a plane. The vector (*u_i_*, *u_j_*, 0)*^T^* is by construction parallel to **Π**_*k*_, then:

(10) $$n_{k}^{T}\left(\begin{array}{c} u_{i}\\ u_{j}\\ 0 \end{array}\right)=0,$$

where **n***_k_* = (*a_k_*, *b_k_*, *c_k_*)*^T^* is the normal to **Π**_*k*_. The three similar equations for the three possible *k* lead to:

(11) Mu=0.

This shows **u** as an element of the kernel of *M*. **u** is not the null vector because C is not reduced to a point, thus *M* is non-invertible and the rank of *M* is not larger than 2. The rank deficiency of *M* means we only have two linearly independent scalar equations from Equation (7), however we can still express two of the velocity components as functions of the last one, e.g., *v*_*x*_:

(12) $$v=\left(\begin{array}{c} v_{x}\\ \frac{-a_{1}v_{x}+c_{1}}{b_{1}}\\ \frac{-b_{3}v_{x}+c_{3}}{a_{3}} \end{array}\right)=v_{x}\underset{q}{\underset{︸}{\left(\begin{array}{c} 1\\ -\frac{a_{1}}{b_{1}}\\ -\frac{b_{3}}{a_{3}} \end{array}\right)}}+\underset{r}{\underset{︸}{\left(\begin{array}{c} 0\\ \frac{c_{1}}{b_{1}}\\ \frac{c_{3}}{a_{3}} \end{array}\right)}},$$

where $Mq=\left(\begin{array}{c} 0\\ \frac{\det(M)}{a_{3}b_{1}}\\ 0 \end{array}\right)=\left(\begin{array}{c} 0\\ 0\\ 0 \end{array}\right).$

This last equation shows that **q** is collinear to **u** if *M* is rank 2, hence we deduce from Equation (7) that *M***r** = (*c*_1_, *c*_2_, *c*_3_)*^T^*.

*Remark 1. *M*'s rank can only be reduced to one if there is no spatial translation. The swept structures in the subspaces defined by (*ijT*) are vertical lines. Such a case is a particular case which is detected when no ruled surface is generated. It does not concern structures undergoing rotations since points not on the rotation axis will have a non null tangential velocity*.

*Remark 2. Expressing **v** as a one parameter vector fails if and only if the rank of *M* is less than 2 i.e., if edges do not generate planes. However, some plane configurations require larger effort to achieve the closed form of **v** such as the case where the plane is perpendicular to one of the spatial frame axes. For example, when the *X*-axis is normal to the plane, Equation (12) is not valid as *b*_1_ and *a*_3_ are equal to zero. This problem can be solved by expressing **v** either as a function of *v*_*y*_ or *v*_*z*_. In that case, we can see that *v*_*x*_ = −*c*_3_/*b*_3_ and *v*_*z*_ is a function of *v*_*y*_. The problem of finding **v** is again reduced to the search for the correct value of one of its component*.

### 2.4. Velocity estimation

**Figure 4:**
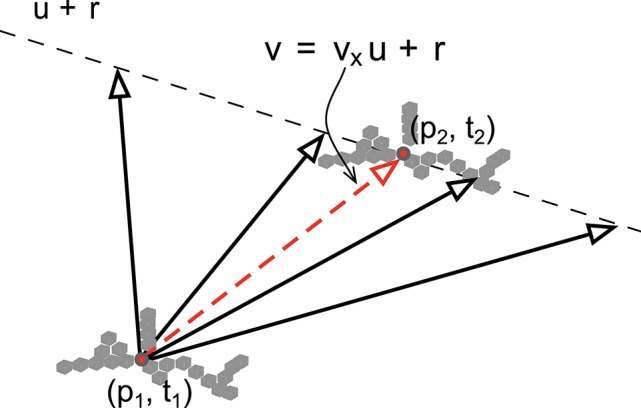
**The velocity is to be determined locally along a line spanned by u and passing by p + r**. This is achieved by matching local structure defined by a set of 3D points (gray cubes in the figure).

As shown in the previous section, from Equation (12), the assumption of local constant velocity motion of straight edges allows to establish a simple linear relation between the velocity vector and the surface swept by the edge points. Estimating the velocity becomes equivalent to identifying the correct real value *v*_*x*_. This is a registration problem for which we need to initiate the point cloud within a spatio-temporal neighborhood as a given structure. We then translate it according to vectors **v**, parametrized by *v*_*x*_. A matching operation is then performed for several sampled values of *v*_*x*_, the correct *v*_*x*_ is the one producing the smallest matching error at the time and location given by the velocity vector (see Figure 4). The procedure to estimate the velocity via the shape registration is explained in detail by Algorithm 1: the search for **v** is now a minimization problem of a error cost function *E*, which is built as explained in the next section.

**Algorithm 1:** **3D flow algorithm**.

**Require:** Stream of 3D events obtained from third-party device/algortihm
1: for **each** 3D event (**p**, *t*) **do**
2: Determine the spatio-temporal neighborhood of 3D event close to (**p**, *t*).
3: Fit 3 planes $\Pi_{1}={\left(a_{1},b_{1},c_{1},d_{1}\right)}^{T},\Pi_{2}={\left(a_{2},b_{2},c_{2},d_{2}\right)}^{T},\Pi_{3}={\left(a_{3},b_{3},c_{3},d_{3}\right)}^{T}$ using a least-square technique to minimize the three scalars:
4:
$$\left|(p_{x},p_{y},t,1)\Pi_{1}|\text{ , }|(p_{x},p_{z},t,1)\Pi_{2}|\text{ , }|(p_{y},p_{z},t,1)\Pi_{3}\right|$$
5: Initialize a large enough interval *R* = [*R*_1_, *R*_*r*_] of length *l* such that ṽ_*x*_ ∈ *R*. Set n = 1.
6: **while** *E* > threshold **and** *n* < max-iteration **do**
7: Divide *R* into *r* intervals *R*_*k*_ of size $\frac{l}{r}$ and define the set {*v*_*k*_} such that *v*_*k*_ is the center of *R*_*k*_.
8: **for** each *v*_*k*_ **do**
9: Compute *E*_*k*_ according to Algorithm 2,
10: **if** *E*_*k*_ is minimal **then**
11: Update *E* ← *E*_*k*_,
12: Update *l* ← *l*(*r* − 1)/*r*,
13: Update $R\leftarrow \left[R_{k}-\frac{l}{2},R_{k}+\frac{l}{2}\right]$.
14: Compute **v**:
$$\text{v}={\left(v_{k},-\frac{a_{1}v_{k}+c_{1}}{b_{1}},-\frac{b_{3}v_{k}+c_{3}}{a_{3}}\right)}^{T}$$
15: Update *n* ← *n* + 1.
16: **end if**
17: **end for**
18: **end while**
19: Return **v**
20: end **for**

### 2.5. Error cost function

A local point cloud centered on the event (**p**1, *t*1)*^T^*, is temporally consistent in the sense that any of its element will be captured at closely the same time. If in addition, the luminance *L* of the events is available, then the cloud local rigidity also ensures that *L* is consistent independently of time. We can therefore state that when the point cloud that moves from **p**_1_ at *t*_1_ to **p**_2_ at *t*_2_, the local geometric structure and the luminance should be preserved. We can formalize the structure matching operation as a minimization of the energy *E* problem and stated as follows:

$$E=E_{S}+E_{T}+E_{L},$$

where *E*_*S*_, *E*_*T*_, and *E*_*L*_ are respectively the geometric, the temporal and the luminance energies. *E*_*T*_ and *E*_*S*_ are minimal as long as the cloud is not deforming when it moves from **p**_1_ to **p**_2_. If the events' brightness is also preserved during this motion then *E*_*L*_ is also minimal.

We define the 3D events cloud *S*(**p**_*i*_, *t*_*i*_) as:

(14) $$\begin{array}{l} S(p_{i},t_{i})=\{q_{j}\in {\mathbb{R}}^{3}|||q_{j}-p_{i}||\;\leq \Delta_{s}\;,t_{j}-t_{i}\leq \Delta_{t}\\ \text{ and }t_{j}>t_{i}\}. \end{array}$$

This set contains all 3D points spatiotemporally close to **p**_*i*_ i.e., points within a neighborhood of **p**_*i*_ of radius Δ_*s*_ in space and length Δ_*t*_ in time. The energy cost associated to each sampled velocity vector for a given point **p**_0_ is computed according to Algorithm 2.

**Algorithm 2:** **Energy cost computation**.

Require: **p**_0_, the set *S*(**p**_0_), *v*_*x*_.
1: Apply Equation 12 with the given *v*_*x*_ to build vector **v**. 2: Define *S*(**p**_0_) + **v**, the translated local structure *S*(**p**_0_) by **v**. Define *S*(**p**_0_ + **v**) the set of points that occur in the neighborhood of **p**_0_ + **v** at *t*_*i*_ + *dt*. 3: With the convention that **p**_*i*_ ∈ *S*(**p**_0_) + **v**, and**q**_*j*_ ∈ *S*(**p**_0_ + **v**), we compute the energy function *E*(**v**) with: (15) $E_{S}=\frac{1}{n}\sum_{i\;=\;1}^{n}\underset{q_{j}}{\min}||p_{i}-q_{j}||,$ (16) $E_{T}=\frac{1}{n}\sum_{i\;=\;1}^{n}\underset{q_{j}}{\min}|t_{i}-t_{j}|.$ where *t*_*j*_ is the time at which **q**_*j*_ occured. 4: Finally, if luminance is available: (17) $E_{L}=\sum_{i=1}^{n}\underset{q_{j}}{\min}|L(p_{i})-L(q_{j})|.$ 5: Return *E*.

*E*_*L*_ is the sum of the smallest luminance difference between all pairs of (**p**_*i*_, **q**_*j*_) and *E*_*S*_ is the mean value of the smallest distances of each **p**_*i*_ to each **q**_*j*_. It is also called the mean closest point between both points clouds and is a dissimilarity measure often used for example in the Iterative Closest Point (ICP) problem (Besl and McKay, 1992). The correct **v** is given by the value *v*_*x*_ which minimizes the energy function *E*:

(18) $${\tilde{v}}_{x}=\underset{v_{x}\in \mathbb{R}}{\text{argmin}}E.$$

More elaborate registration techniques to track deformable 3D surfaces may be used for this matching operation. We can mention the most notable ones, Starck and Hilton (2007), Ahmed et al. (2008), and Zeng et al. (2010), that are not using any shapes prior. Accurate registrations are achieved by combined use of several surface features, followed by a coarse to fine scheme. These techniques are however not suitable in their actual form for processing textureless and event-based inputs.

To minimize *E* with respect to *v*_*x*_, we also applied a dichotomic search strategy to sample possible values of *v*_*x*_ and match local 3D structure accordingly. Let *R* = [*R*_1_, *R*_*r*_] be a real interval that is set large enough at the beginning of the search to make sure it contains ṽ_*x*_. Fixing *R* large enough is only necessary when no recent past estimations of the velocity have been calculated at **p**_*i*_, otherwise the length of *R* is defined from the previous estimation of *v*_*x*_. To determine precisely ṽ_*x*_, *R* is subdivided into *r* equal length intervals and the centers of all intervals give a set of possible values for *v*_*x*_. The error cost function is computed for each *v*_*x*_ and the interval producing the smallest *E* is used to update *R*.

This operation is iterated until *E* is below a preset threshold and after a minimum number of iterations. This threshold is defined experimentally with the purpose of optimizing the structure matching process by limiting the search to an acceptable matching error. This threshold can be related to the point cloud density and if it is set to zero, then the maximum number of matching iterations is always performed. *r* is usually set to 5, however it can be larger. Estimation accuracy increases with *r* but at the cost of longer processing time.

### 2.6. Optimal spatiotemporal neighborhood

The correct estimation of the velocity is conditioned by the spatiotemporal neighborhood, defined as the spatiotemporal volume of dimensions (Δ_*x*_×Δ_*y*_×Δ_*z*_×Δ_*t*_), in which the 3D point cloud has moved from time *t* to *t* + *dt*. A large neighborhood will allow to find the correct match, but at the cost of processing a large set of data, on the contrary, a too small one will not allow to match the local structures. The spatiotemporal neighborhood must also be resized automatically and dynamically in accordance to the 3D points' velocity. In our implementation, we deal with the problem by adjusting a linear function on the neighborhood size e.g., **s**_*k*_ = (Δ*_x_*, Δ*_y_*, Δ*_z_*, Δ*_t_*)*^T^* is a linear combination of the m previous values **s**_*k*−1_, …, **s**_*k*−*m*_:

(19) $$s_{k}=\sum_{i=1}^{m}a_{i}s_{k-i},$$

where the coefficients *a*_*i*_ are estimated with a standard linear prediction coding scheme (Durbin, 1959). The value of *m* is usually set to 5 according to experimental results while the initial value **s**_0_ is deduced from the coarse estimation of the initial velocity i.e., the mean translation between the first two frames. Thus, we have $s_{0}={\left(v_{0}dt,dt\right)}^{T}$, assuming **v**_0_ is the initial estimate of the velocity.

The asynchronous 3D flow extraction from points clouds can be achieve by implementing Algorithms 1, 2 and improved if necessary with the optimal neighborhood estimation. This proposed approach does not require clusters of 3D points captured at the same *t* as one uses to have with frame-based reconstructions, yet it can still be applied if the inputs are frame-based.

## 3. Results

The first set of experiments are performed on synthetic scenes, where both 3D structures and motion (velocity and trajectory) are known. These results measure the theoretical performance (without noise or reconstruction errors) of our method through comparison between estimated velocity vectors and the known motion.

The second set of experiments are performed on natural scenes, with the purpose of showing the performance of the event-based fitting method when dealing with real data. The algorithm is applied to two sources of 3D data: a Microsoft Kinect (an RGBD sensor that outputs frames of 3D points aligned with RGB information) and an asynchronous event-based 3D reconstruction system as introduced in Carneiro et al. (2013).

### 3.1. Simulated scenes

**Figure 5:**
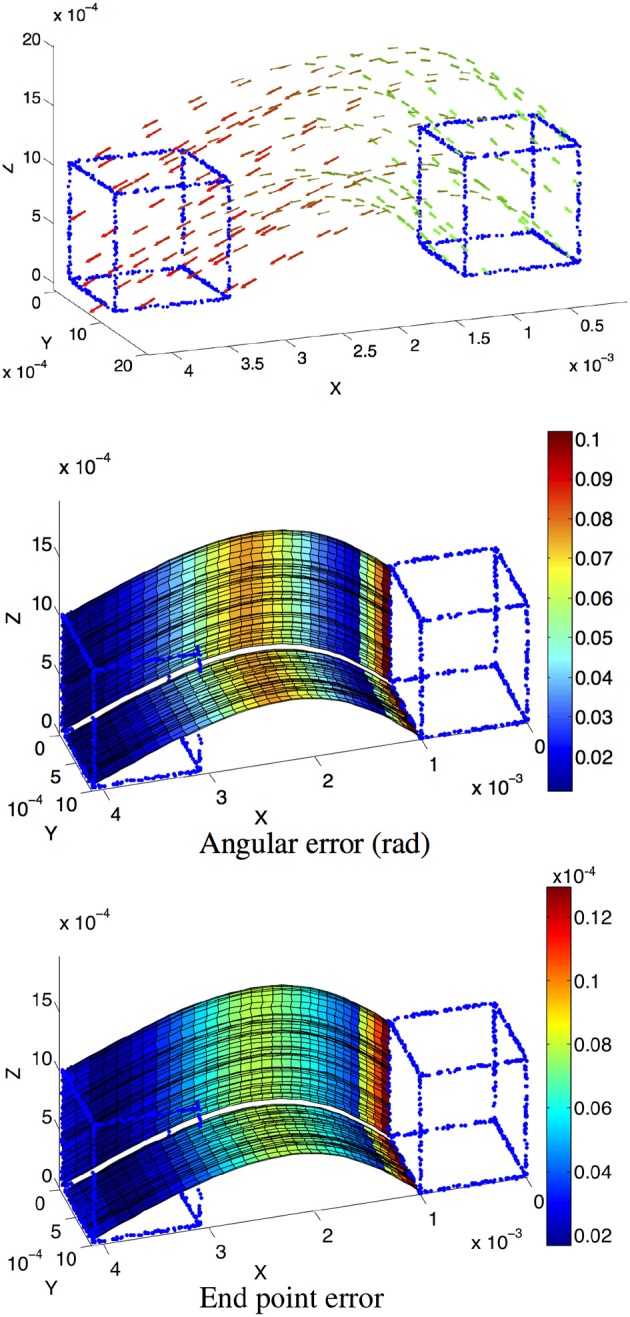
**(Top)** Scene flow of a cube with the color coding time, as the cube moves from right to left. **(Center and Bottom)** Angular and endpoint errors of the estimated velocity field. The patches of planes are underlined to show the locally constant velocity assumption. For visibility purpose, the velocity is only shown for two edges. All axes are expressed in length unit except for the angle color scale.

**Figure 6:**
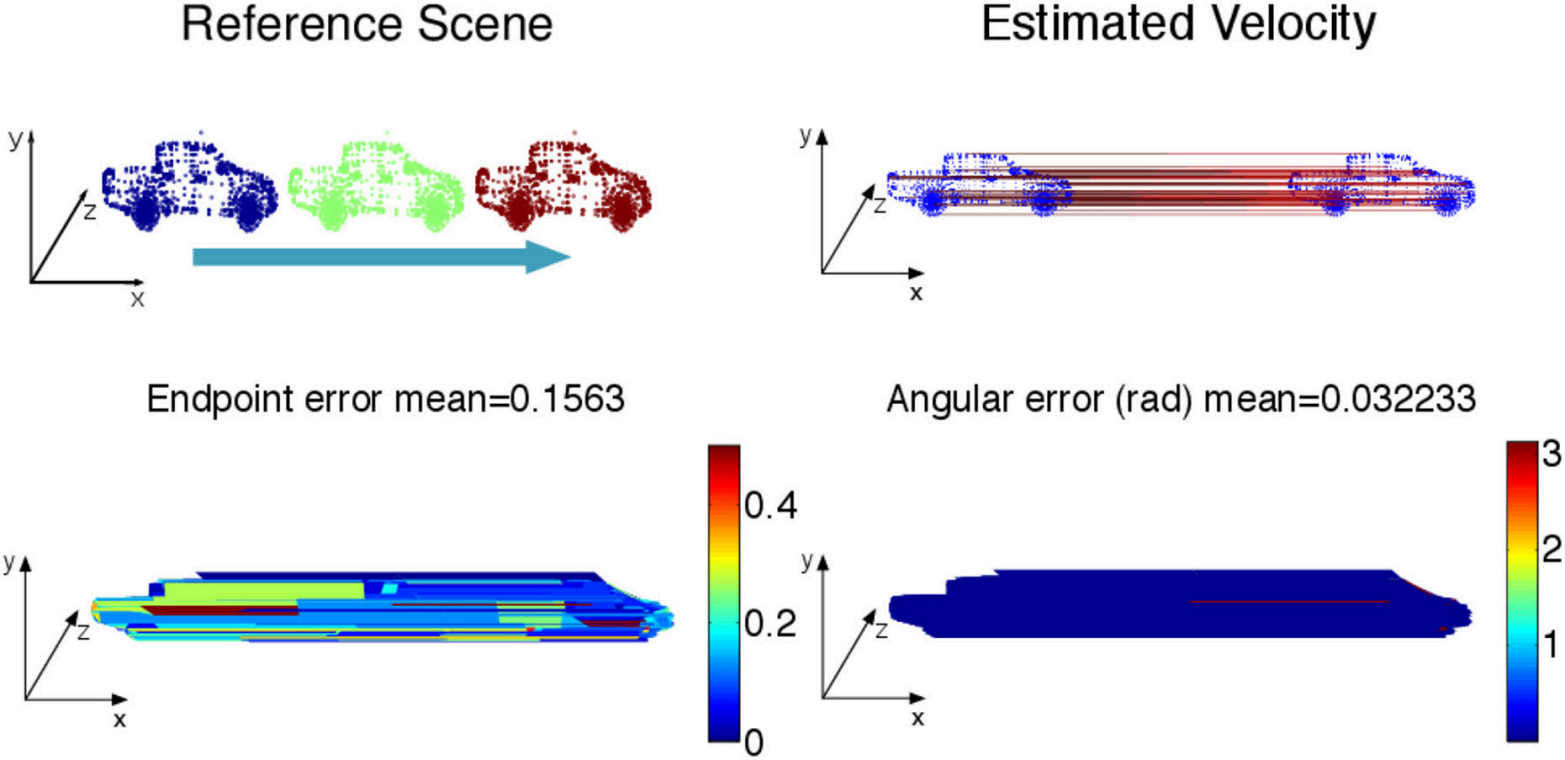
**(Top)** Constant velocity translation with the color coding time, as the car moves in a straight line from left to right at 10*m*/*s*. The estimated velocity is shown on the right. **(Bottom)** Angular and endpoint errors of the estimated velocity field with the 3D flow technique.

**Figure 7:**
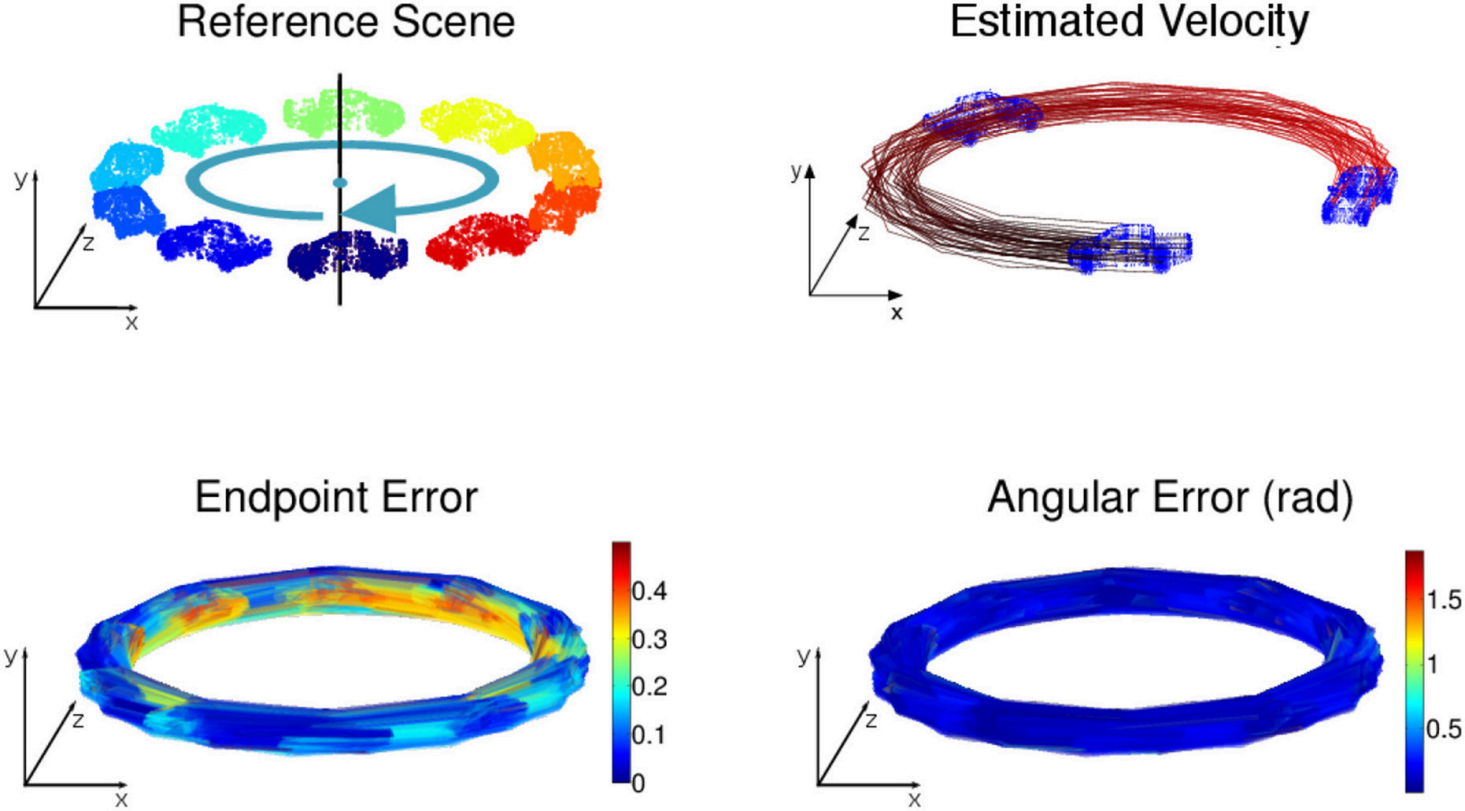
**(Top)** Circular motion with no tangential acceleration: the car's trajectory is outlined via the velocity estimation. **(Bottom)** Angular and endpoint errors of the estimated velocity field.

**Figure 8:**
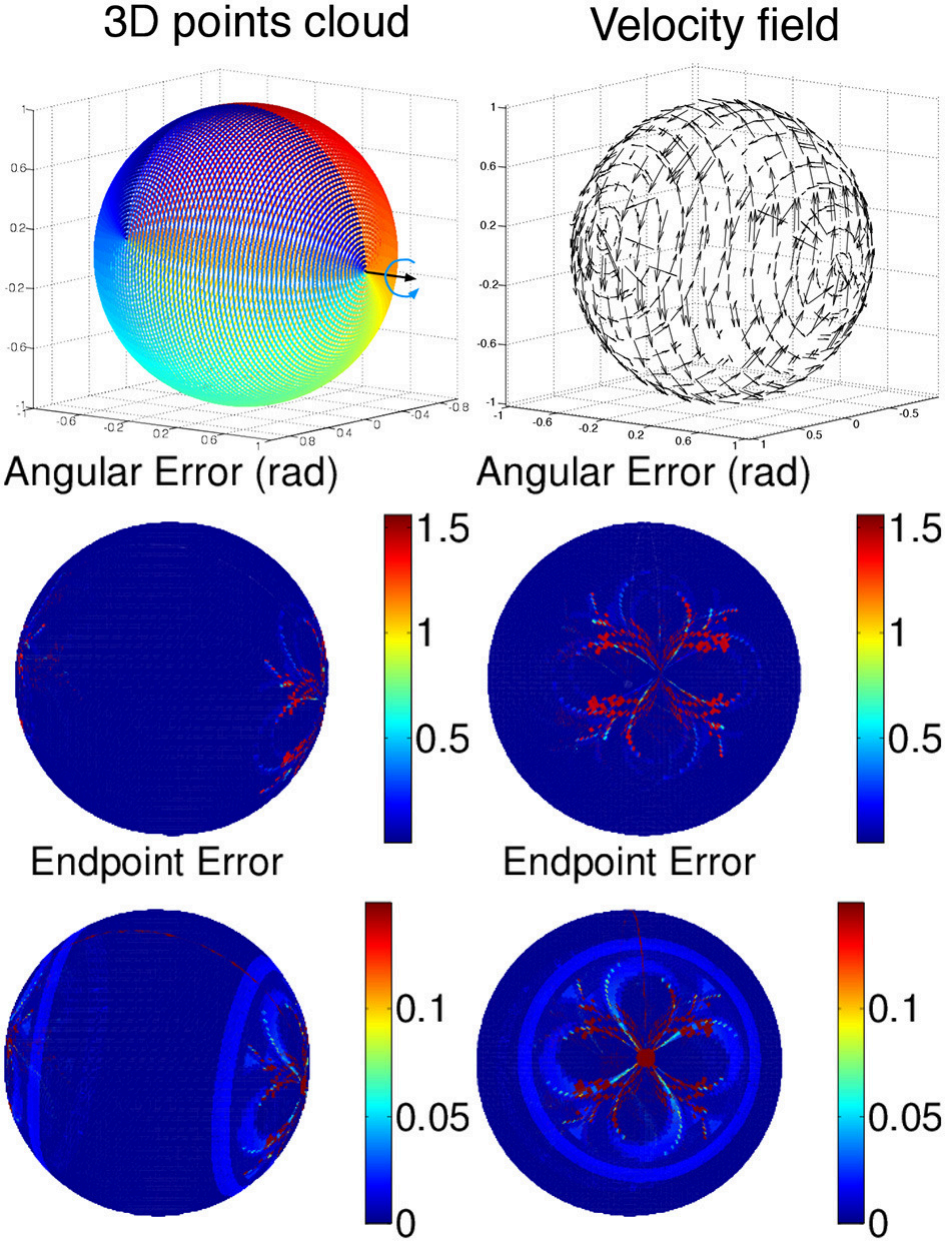
**Scene flow of a rotating sphere. (Top)** Time color coded representation of the sphere and the velocity field are represented on the top row. Angular and endpoint errors of the estimated velocity field are shown for two points of view. Error are shown in absolute values: the endpoint errors are not exceeding 0.1 length unit in the dark blue regions and is higher as we are close to the poles because of the high density of samples for which the fitting parameters are no optimal enough.

Four simulated scenes are synthetized: (Figure 5) a smooth translation of a wire cube at constant amplitude; (Figure 6) a 3D car model undergoing a straight translation at 10 m per second; (Figure 7) the same 3D car model describing a circular motion; (Figure 8) a pure rotation of a sphere at constant angular speed.

For each scene, the velocity flow is computed using the geometric structure information alone (only the 3D points' positions and timestamps are given in the simulation). The energy cost function in Algorithm 2 is reduced to *E*_*s*_. The flow performance is measured by two quantities conventionally used to validate optical flow, the angular error, which is the angle defined by the estimated normalized velocity vector $\tilde{\text{v}}$ and the ground-truth **v**. The angle is given by the inverse cosine of the scalar product of 2 vectors:

(20) $$\arccos\left({\tilde{v}}^{T}v/|\tilde{v}||v|\right).$$

This measure has been introduced in Fleet and Jepson (1990) to assess the accuracy of the flow direction. However, the angular error can be biased by large velocity vectors for which the differences in amplitude can be significant and in the same time, the angular errors are small. In that sense the angular error is favoring the large motion over the smaller ones. To compensate for that bias, a second performance measure, the endpoint error, introduced by Otte and Nagel (1994) is conjointly used. This endpoint error is the norm of the difference between the estimated velocity and the real one:

(21) $$|v-\tilde{v}|.$$

Both estimated angular error and endpoint error are represented with a color scaled representation (Figures 5–**15**). For the moving cube, the maximal error occurs at the beginning of the motion and is due to the fitting spatio-temporal neighborhood, chosen as the best compromise for the entire motion.

The results on synthetic data, summarized in Table 1, show the ability of the method to estimate densely and smoothly the velocity field. The rotating sphere is a challenging because the 3D points composing the surface are not spatially uniform. The non uniform acceleration on the sphere also implies non uniform tangential velocity of 3D points on which we fit the local planes. This explains why the velocity estimation is slightly less accurate for rotations. For translations (cube and car), the velocity is estimated with higher accuracy since the direction has a mean angular error of 0.04 *rad* (with a peak value of 0.1 *rad*) and a mean endpoint error of 0.8%, with a peak value of 1.2% when normalized by the ground-truth objects sizes (respectively the cube edge length, the sphere diameter, and the length of the car). For rotations, the accuracy has the same order of magnitude: around 0.15 *rad* and 2.2% for respectively the angular and the endpoint error.

**Table 1:** **Average motion estimation errors for the synthetic scenes**.

	**Cube translation**	**Car cst. translation**	**Car circular motion**	**Rotating sphere**
Angular error (rad)	0.04	0.03	0.15	0.15
Endpoint error (%)	0.8	1.5	2.2	2

### 3.2. Natural scenes

The second set of results is obtained from real scenes showing a moving person in the scene. The 3D point clouds are provided by a Kinect sensor that also measures the RGB intensity. The Kinect provides depth information for every detected pixel. The background pixels representing the room's walls are removed via depth segmentation. In these sequences, the person is a nice example of a deformable target with limbs moving at different non-constant velocities. However, the local constant speed hypothesis holds. It is sufficient to allow a smooth estimation of the scene flow. Scene flows estimations are given as two sets of results. The first one uses only geometric constraints, when the scene luminance is not available for the structure registration operation. The second set uses the additional information brought by the luminance in addition to the geometry.

The flow estimation for each sequence is assessed in two ways:

1. A reference speed is established using the person's head to compute speed across frames. The head's position at time *t* is annotated manually to build a reference motion scene. This is then used as ground-truth to evaluate the event-based fitting method.
2. If *S*(*t*) designates an arbitrary point cloud in the scene at time *t* then *S*(*t*)+**v***dt* is the morphing of *S*(*t*) by the translation vector **v***dt*.
  Let **p**_*i*_ ∈ *S*(*t*) and **q**_*i*_ ∈ *S*(*t*) + **v***dt* such that:
  (22) $$q_{i}=\underset{q\in S(t)+vdt}{argmin}||q-(p_{i}+vdt)||.$$
  We define the morphing error as the mean error of each pair (**p**_*i*_, **q**_*i*_):
  (23) $$\frac{1}{N}\sum_{i}||p_{i}-q_{i}||.$$
  This morphing error, normalized by the mean ground-truth velocity amplitude (provided by tracking limbs from the Kinect output for the 3 sequences), is used as the second performance measurement for the rest of the paper.

#### 3.2.1. First sequence

**Figure 9:**
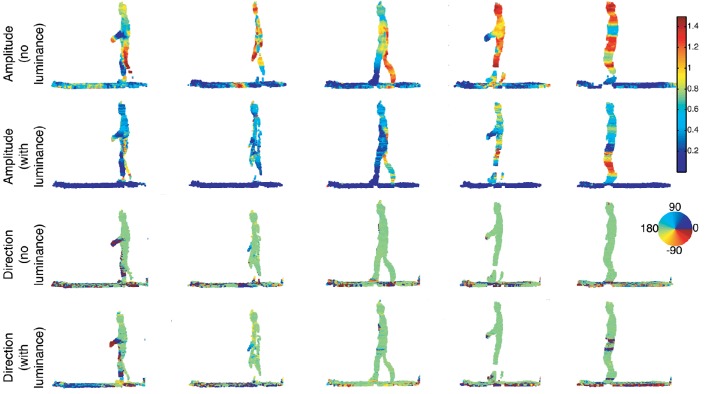
**Sequence of a person walking at constant speed across the scene**. The amplitude of each 3D point is color-coded and shows that the event-based plane fitting technique is able to estimate non rigid object velocity without and with luminance. A color scale is also used for the flow directions. One can see the person going from the right to the left as confirmed by the color (green in the color scale i.e., an angle of 180°). Again, we can observe that directions can be accurately estimated by using only time and geometry. The addition of luminance provides slight improvements.

In the first sequence, shown in Figure 9, a person walks in front of the cameras at a constant pace. The velocities' amplitudes, and the directions are shown separately for both estimations without using luminance information. These figures show the performance of the algorithm in the presence of a deformable object. The limbs, in particular, the legs and the fingertips which are subject to the largest velocity changes show clear phases of acceleration: when the legs reach the end of the step, the speed is close to zero (1st and 3rd images), it reaches a maximal value when the legs are in the middle of the step (5th image). The velocity changes are also visible in the color coded motion directions: the silhouettes are not all green as the hands swing. The floor, as it is scanned by the Kinect sensor, was also processed by the algorithm. The estimated speeds are largely coherent with what it is expected: they are close to zero, thus negligible with respect to the moving person. The measured velocity variation (in amplitude and direction) from the floor are mainly due to several sources of noise coming from the sensor itself, the lighting change induced by the motion, etc. The background wall has been removed using depth information before the scene flow estimation is applied. This eliminates any non relevant events/pixel changes due to shadows.

In this experiment, the person walks across the scene, in front of the cameras at a constant speed of 1*m*/*s*. This reference speed is measured by manually segmenting the head's point cloud for each frame. The speed is also extracted for the head from the estimated 3D flow with Algorithm 1. The top row of **Figure 11** shows both speed curves, plot together. Square markers represent the reference speed, circle markers show the speed estimated without luminance information while the diamond markers represent the result achieved with the luminance (through the term *E*_*L*_ in Equation 13). The speed estimated from the geometric constraint has a mean value of 0.99*m*.*s*^−1^ and the one using luminance is around 1.2*m*.*s*^−1^. The relative mean difference between the two estimations is around 17%. This shows that both estimates are coherent.

The small fluctuations of the estimated speed are not surprising as the trajectory of the head is not a straight translation: body weight transfer happens at each step and it modifies subsequently the head velocity in amplitude and direction. Finally, the color coded flow directions are consistent. Results show that the flow is pointing at 180°, i.e., from right to left for most of the body except for the person's hands. Floor's directions however have a random distribution. We can explain this result by two causes: noise in the acquired data as the floor is a matt surface diffusing randomly the neon lighting and the shape registration procedure in the algorithm which is unlikely to register correctly structures on a uniform surface.

#### 3.2.2. Second sequence

**Figure 10:**
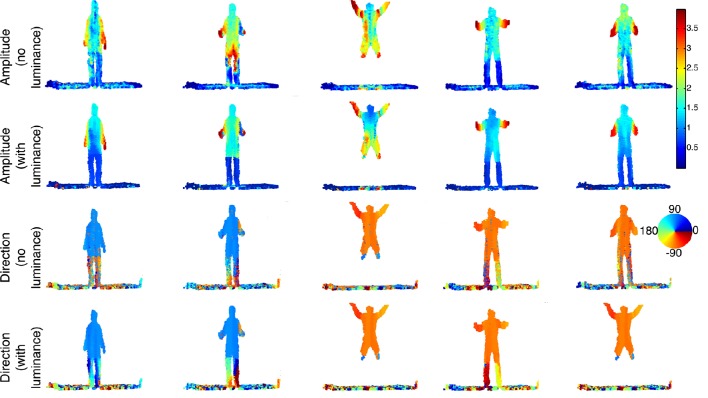
**Sequence of a jumping person**. This is a complex motion which comprises several rapid changes of the velocity in direction and amplitude. The amplitude plot of the velocity for each 3D points is color-coded. Parts of the body can be segmented according to the velocity e.g., the arms, the legs and rest of the body which have distinctive amplitude. The color coded flow directions (expressed in degree) are well estimated as we can see for the whole body, the direction is pointing up (i.e., angle close to 90°) and pointing to the bottom when the person is falling (i.e., angle around −90°).

**Figure 11:**
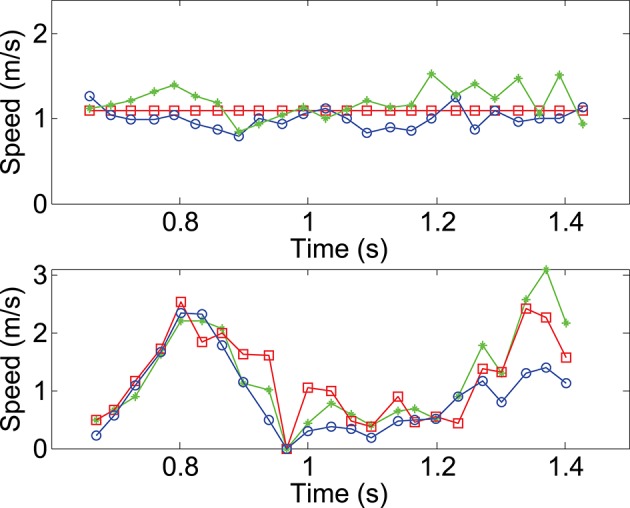
**Mean velocity computed for the head in the walking (top)** and the jumping **(bottom)** sequences. The circle curves are estimations achieved by time and geometric information. The diamond curves are results one gets when luminance is used for the structure registration. Finally, the square curve represents the velocity of the manually segmented head's 3D points.

In the second sequence (Figure 10), a more complex motion is tested, showing a person jumping. The velocity amplitude changes several times throughout the sequence: it increases at the beginning and reaches a maximum, then decreases to 0 when the person is at the top of its trajectory. The amplitude then increases again during the fall until he reaches the ground. This sequence of speed change is shown at the bottom row of Figure 11. Similarly to the walking sequence, both reference speed curves and estimation are shown together. However, in this experiment, it is more difficult to assess the accuracy of the estimation since the reference speed itself is built with a low accuracy. This is due to the difficulty to manually segment the head's 3D point since the speed changes too quickly.

The jumping sequence is an ideal example of a non-rigid body moving at a totally unconstrained speed. The arms, in particular, show the largest velocity changes since the person swings them to gather momentum from the first half of the jump and he folds them back once the body begins to fall. In this sequence one can also observe the velocity estimated for the floor which is again mostly equal to zero, except at the right under the jumping point because of the moving shadow of the person. The velocities are pointing mainly up (i.e., angle of 90°) during the ascending phase and pointing down when he is falling (i.e., angle of −90°).

For both walking and jumping sequences, a higher accuracy is achieved in estimating the velocity when luminance is used, as shown in Table 2 for five frames taken from the sequences. The mean morphing error is below 3% for the walking sequence and slightly higher than 2% for the jumping one when luminance information is used. The estimation performance is slightly lower when the luminance is removed. In these cases, the morphing errors increase respectively to 5 and 7%. Two main observations should be retained from these results: first, morphed point clouds still consist of well defined objects. This shows the computed motion is consistent for the full scene as morphing objects do not produce incoherent shapes. Secondly, the estimated scene flow is shown being consistent with the real motion since *S*(*t*)+**v***dt*, the morphed point cloud, matches correctly *S*(*t* + *dt*), the point cloud at *t*+*dt*.

**Table 2:** **Morphing error for several sets of five randomly selected times in the sequences**.

**Morphing error (ratio)**				
**Frame**	**Walking**		**Jumping**	
	**Without ***L*****	**With ***L*****	**Without ***L*****	**With ***L*****
1	0.0542	0.0214	0.0250	0.0131
2	0.0695	0.0277	0.0635	0.0270
3	0.0786	0.0221	0.0698	0.0414
4	0.0863	0.0424	0.0510	0.0128
5	0.0433	0.0278	0.0481	0.0143
Mean	0.0664	0.0283	0.0515	0.0217

Errors are measured with and without luminance information. In the first case, the mean error are around 6.6 and 5.2% respectively. These ratios are improved to 2.8 and 2.2% when luminance is added i.e., errors are reduced by a factor close to 2.

### 3.3. 3D point clouds from event-based vision sensors

**Figure 12:**
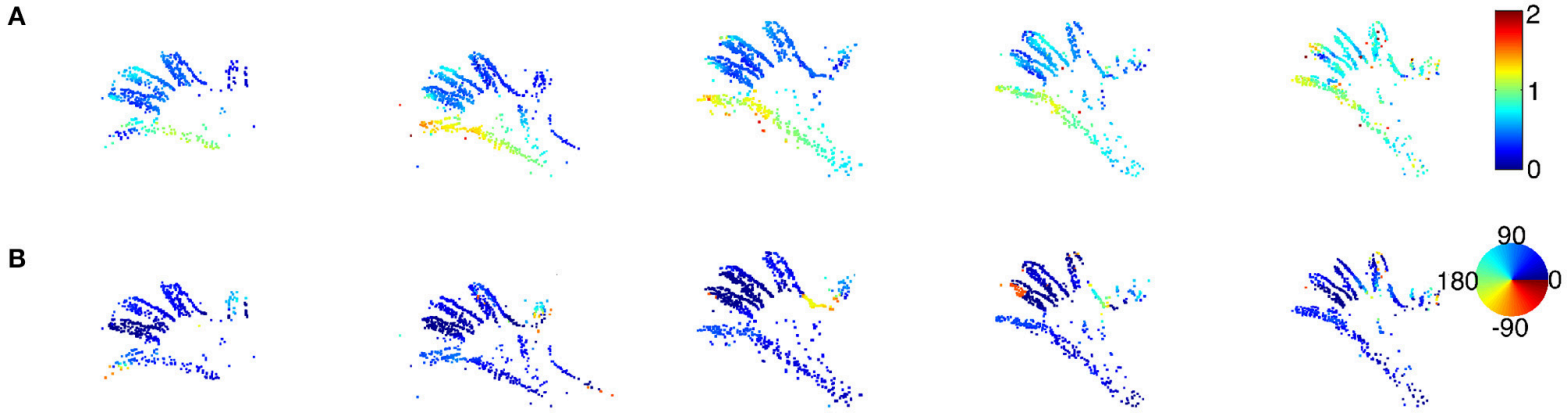
**A sequence of a moving hand acquired using a stereo-rig composed of event-based silicon retinas (DVS) as sets of event {(p, *t*)}**. No luminance information is available from these sensors. In **(A,B)** color-coded plots of the estimated amplitude and direction of the velocity for each reconstructed 3D points. In contrast with the previous resultsare shown, they correspond to the locations of dense 3D points. The motion is a smooth translation from left to right as shown by the direction plots pointing toward 45°.

**Figure 13:**
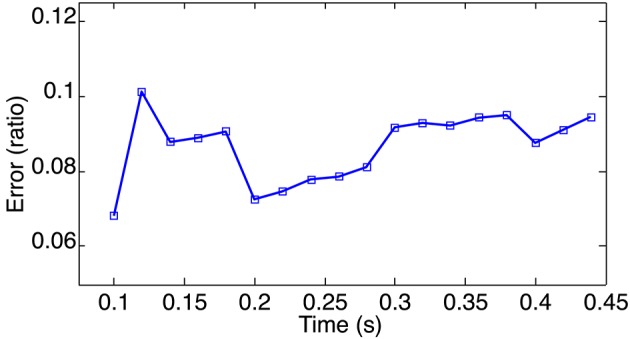
**Morphing error for the event-based 3D point clouds of a waving hand calculated for a set of randomly selected times**. The Mean error is around 8.7%, this is consistant with previous estimations if no intensity information is used or available.

This subsection provides the 3D scene flow using event-based cameras (DVS) as described in Carneiro et al. (2013). Computed 3D data have a high resolution of 1μ*s*. The input to the scene flow estimation are asynchronous 3D point clouds of a hand closing and opening in front of the stereo rig, while the second sequence is a moving face captured by the same stereo rig. The hand speed is of the order of one meter per second while the face moved slower (several cm per second). The first sequence's results are shown in Figures 12, 13. The events generated by the hand's contours are sufficient to estimate 3D flow estimation. The direction and amplitude are consistent with the motion. The mean morphing error, (8.7%), is at the same order of magnitude than the previous experiments.

**Figure 14:**
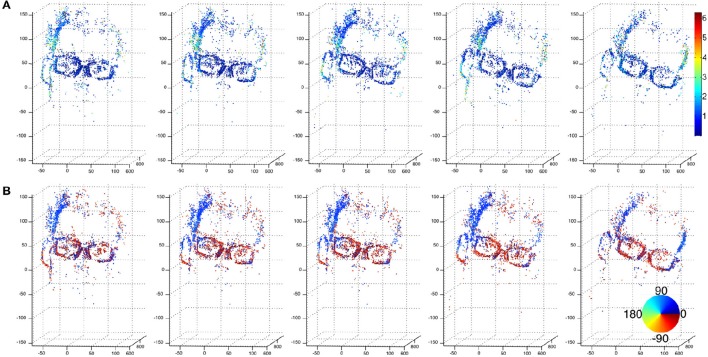
**Sequence of a moving face 3D point clouds computed using event-based cameras**. **(A)** Shows the color coded flow's amplitude while **(B)** is its color coded directions in a fronto parallel plane. The computed flow outlines the actual motion of the face that rotates from right to left: temples motion is pointing to 30° (blue) while the glasses motion is almost horizontal and pointing to the right (dark red). The global motion is close to be constant over the samples shown by the figure. The amplitude is expressed in cm/s.

The second 3D scene flow estimation from the event-based cameras are shown without providing morphing error for readability reason. The second sequence shows a face moving in front of the event-based cameras. The color-coded flow are shown in Figure 14 with row (a) color-coding the flow's directions while (b), shows the motion amplitudes.

### 3.4. Comparison to the particle filter scene flow estimation

This third set of results obtained with the event-based plane fitting technique (without luminance information) is compared to the method published in Hadfield and Bowden (2014). This paper models a set of moving 3D points using a particle filter that supports multiple motion hypotheses to estimate the 3D scene flow from the 3D points provided by a Kinect.

**Figure 15:**
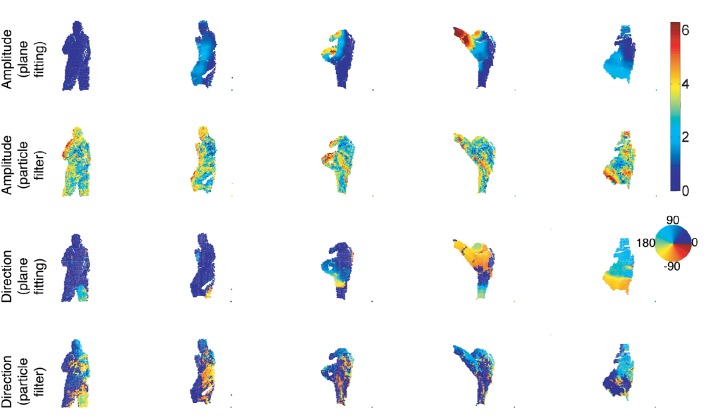
**Sequence of a person performing a kick**. The amplitude of each 3D point is color-coded and shows respectively the flow amplitude estimation using the event-based plane fitting and the particle filter method proposed in Hadfield and Bowden (2014). The flow direction estimation from the event-based plane fitting and the particle filter methods are shown in the 2 last lines.

The sequence and its estimated scene flow presented in Hadfield and Bowden (2014) were kindly provided by the authors and are shown as output without additional processing. Figure 15 shows samples of this sequence along with the estimated 3D scene flow: the velocity amplitude and direction are shown in two separate color-coded representations for the two methods. One can notice that the event-based plane fitting method produces smoother results that are consistant with the scene content, especially the velocity is expected to be maximal at the foot when the kick is accomplished.

We provide the morphing error in Table 3. It shows the error computed for 5 frames taken at some arbitrary regular time interval, using both method. This is consistant with the previous analysis showing that the event-based plane fitting method's performance is higher than the state-of-the-art frame-based technique. Unsurprisingly, the overall estimation accuracy for both methods is lower than the previous experiments mainly because the Kinect's inability to accurately capture fast motions that give rise to blurry images.

**Table 3:** **Event-based plane fitting technique compared to particle filtering technique**.

**Morphing error (ratio)**		
**Frame**	**Plane fitting**	**Scene particle**
1	0.0124	0.0265
2	0.0382	0.0461
3	0.0496	0.0505
4	0.0344	0.0378
5	0.0436	0.0480
Mean	0.0356	0.0418

*Errors are normalized by the mean velocity of the left leg*.

## 4. Discussion

This paper introduced a new technique of dense 3D scene flow estimation. This is so far, the first 3D scene flow algorithm developed for asynchronous sensing using event-based cameras. The event-based formulation of the flow applies the rule of “one event equals one computation” that allows an incremental update of the 3D scene flow in an almost continuous manner. This formulation allows also a straightforward extension to frame-based representation as long as time is used as the main computation feature.

The motion inference and 3D reconstruction from multiple cameras are usually coupled tasks in frame-based computer vision. They are solved by stereovision mechanisms which require highly accurate calibration operation. Conventionally, the dense scene flow is estimated and refined from the dense optical flow which is its projection on the image planes in an iterative feedback loop scheme. The scene flow computation is therefore a complex problem which is an optimization problem under several conflicting constraints.

The proposed technique is based on the local constant motion of the 3D point clouds and on their locally non deformable geometry. These hypotheses, when satisfied, tell us that an object moving through space, locally generates ruled surfaces from which the velocity vectors can be extracted. The solution we proposed is simple as it constraints the 3D velocity estimation to a search for a parametrization value over the set of real numbers. To achieve this search, we developed a local 3D structure matching strategy using the geometric consistency and when it is available, luminance as an additional constraint to identify structures across time. Experimental results obtained from synthetic and natural scenes show the technique to be particularly suitable in estimating the velocity vectors of deformable objects, undergoing arbitrary unconstrained motions. This approach allows flow estimation from any data output by sensors that capture the spatiotemporal information that but do not necessarily provide nor use luminance such as range finders (e.g., the LiDAR).

The method also provides a dense estimation of the velocity field as an alternative method to using a variational formulation (a very powerful but also highly computationally demanding technique) for flow estimation. Since the plane fitting we applied for the flow regularization is relatively inexpensive operation, the resources are mainly needed for the structure matching operation. We can sketch an idea about the complexity of that matching operation according to Algorithm 1:

- There are 2 nested loops (line 6 and 8 in the algorithm), if we assume that the while loop is satisfied in *n* iterations and that we have *n* values of *v*_*k*_ to test in the for loop, then the algorithm is at least in $O\left(n^{2}\right)$.
- In the case of a stream of *z* events, the complexity is in $O\left(z.n^{2}\right)$.

From that perspective, the complete scene flow algorithm‘ complexity is at least in $O\left(z.n^{2}\right)$. We can reasonably state that this is still less complex compared to conventional scene flow estimation techniques based on particular filter or variational approaches.

## Author contributions

SI: drafting the work and revised the final version for submission. JC: conception and design of the work; data acquisition and analysis; drafting the paper. RB: drafting the work and revising it critically.

### Conflict of interest statement

The authors declare that the research was conducted in the absence of any commercial or financial relationships that could be construed as a potential conflict of interest.
